# New Truths in Ophthalmology

**Published:** 1896-10

**Authors:** 


New Truths in Ophthalmology as developed by G. C. Savage, M. D., Professor of Ophthalmology in the Medical Department of Vanderbilt University, Ex-President of the Nashville Academy of Medicine, President of the Tennessee State Medical Society, Member of Eighth International Congress of Ophthalmology. With fifty-eight illustrations, 265 pages, 12mo, third edition. Price, $2.00. Nashville, Tenn.: Published by the Author. 1896.

The popularity of this little work is evinced by its having reached the third edition. This edition contains several new chapters which will materially increase the demand for the work, as they treat of subjects of much interest, but of great perplexity, to the ophthalmologist. These new chapters include, The functions of the oblique muscles, especially as they are related to oblique astigmatism ; Obliquity of retinal images in oblique astigmatism, as shown by photography ; Sthenic and asthenic orthophoria and heterphoria; Presbyopia deferred by rhythmic exercise of the ciliary muscle.

The author's views, while not fully endorsed by all, are well worthy of consideration and study. Their fuller development in this edition will be welcome. A. A. H.

